# Validation of predicted mRNA splicing mutations using high-throughput transcriptome data

**DOI:** 10.12688/f1000research.3-8.v2

**Published:** 2014-04-07

**Authors:** Coby Viner, Stephanie N. Dorman, Ben C. Shirley, Peter K. Rogan

**Affiliations:** 1 Department of Computer Science, University of Western Ontario, London, Ontario, N6A 5B7, Canada; 2 Department of Biochemistry, University of Western Ontario, London, Ontario, N6A 5C1, Canada; 3 Cytognomix, Inc., London, Ontario, N6G 4X8, Canada

## Abstract

Interpretation of variants present in complete genomes or exomes reveals numerous sequence changes, only a fraction of which are likely to be pathogenic. Mutations have been traditionally inferred from allele frequencies and inheritance patterns in such data. Variants predicted to alter mRNA splicing can be validated by manual inspection of transcriptome sequencing data, however this approach is intractable for large datasets. These abnormal mRNA splicing patterns are characterized by reads demonstrating either exon skipping, cryptic splice site use, and high levels of intron inclusion, or combinations of these properties. We present, Veridical, an *in silico* method for the automatic validation of DNA sequencing variants that alter mRNA splicing. Veridical performs statistically valid comparisons of the normalized read counts of abnormal RNA species in mutant versus non-mutant tissues. This leverages large numbers of control samples to corroborate the consequences of predicted splicing variants in complete genomes and exomes.

## Introduction

DNA variant analysis of complete genome or exome data has typically relied on filtering of alleles according to population frequency and alterations in coding of amino acids. Numerous variants of unknown significance (VUS) in both coding and non-coding gene regions cannot be categorized with these approaches. To address these limitations, *in silico* methods that predict biological impact of individual sequence variants on protein coding and gene expression have been developed, which exhibit varying degrees of sensitivity and specificity ^1^. These approaches have generally not been capable of objective, efficient variant analysis on a genome-scale.

Splicing variants, in particular, are known to be a significant cause of human disease ^2– 5^ and indeed have even been hypothesized to be the most frequent cause of hereditary disease ^6^. Computational identification of mRNA splicing mutations within DNA sequencing (DNA-Seq) data has been implemented to varying degrees of sensitivity, with most software only evaluating conservation solely at the intronic dinucleotides adjacent to the junction (i.e. ^7^). Other approaches are capable of detecting significant mutations at other positions with constitutive, and in certain instances, cryptic, splice sites ^5, 8, 9^ which can result in aberrations in mRNA splicing. Presently, only information theory-based mRNA splicing mutation analysis has been implemented on a genome scale ^10^. Splicing mutations can abrogate recognition of natural, constitutive splice sites (inactivating mutation), weaken their binding affinity (leaky mutation), or alter splicing regulatory protein binding sites that participate in exon definition. The abnormal molecular phenotypes of these mutations comprise: (a) complete exon skipping, (b) reduced efficiency of splicing, (c) failure to remove introns (also termed intron retention or intron inclusion), or (d) cryptic splice site activation, which may define abnormal exon boundaries in transcripts using non-constitutive, proximate sequences, extending or truncating the exon. Some mutations may result in combinations of these molecular phenotypes. Nevertheless, novel or strengthened cryptic sites can be activated independently of any direct effect on the corresponding natural splice site. The prevalence of these splicing events has been determined by ourselves and others ^5, 11– 13^. The diversity of possible molecular phenotypes makes such aberrant splicing challenging to corroborate at the scale required for complete genome (or exome) analyses. This has motivated the development of statistically robust algorithms and software to comprehensively validate the predicted outcomes of splicing mutation analysis.

Putative splicing variants require empirical confirmation based on expression studies from appropriate tissues carrying the mutation, compared with control samples lacking the mutation. In mutations identified from complete genome or exome sequences, corresponding transcriptome analysis based on RNA sequencing (RNA-Seq) is performed to corroborate variants predicted to alter splicing. Manually inspecting a large set of splicing variants of interest with reference to the experimental samples’ RNA-Seq data in a program like the Integrative Genomics Viewer (IGV) ^14^, or simply performing database searches to find existing evidence would be time-consuming for large-scale analyses. Checking control samples would be required to ensure that the variant is not a result of alternative splicing, but is actually causally linked to the variant of interest. Manual inspection of the number of control samples required for statistical power to verify that each displays normal splicing would be laborious and does not easily lend itself to statistical analyses. This may lead to either missing contradictory evidence or to discarding a variant due to the perceived observation of statistically insignificant altered splicing within control samples. In addition, a list of putative splicing variants returned by variant prediction software can often be extremely large. The validation of such a significant quantity of variants may not be feasible, for example, in certain types of cancer, in instances where the genomic mutational load is high and only manual annotation is performed. We have therefore developed Veridical, a software program that automatically searches all given experimental and control RNA-Seq data to validate DNA-derived splicing variants. When adequate expression data are available at the locus carrying the mutation, this approach reveals a comprehensive set of genes exhibiting mRNA splicing defects in complete genomes and exomes. Veridical and its associated software programs are available at: www.veridical.org.

## Methods

The program Veridical was developed to allow high-throughput validation of predicted splicing mutations using RNA sequencing data. Veridical requires at least three files to operate: a DNA variant file containing putative mRNA splicing mutations, a file listing of corresponding transcriptome (RNA-Seq) BAM files, and a file annotating exome structure. A separate file listing RNA-Seq BAM files for control samples (i.e. normal tissue) can also be provided. Here, we demonstrate the capabilities of the software for mutations predicted in a set of breast tumours. Veridical compares RNA-Seq data from the same tumours with RNA-Seq data from control samples lacking the predicted mutation. However, in principle, potential splicing mutations for any disease state with available RNA-Seq data can be investigated. In each tumour, every variant is analyzed by checking the informative sequencing reads from the corresponding RNA-Seq experiment for non-constitutive splice isoforms, and comparing these results with the same type of data from all other tumour and normal samples that do not carry the variant in their exomes.

Veridical concomitantly evaluates control samples, providing for an unbiased assessment of splicing variants of potentially diverse phenotypic consequences. Note that control samples include all non-variant containing files (i.e. RNA-Seq files for those tumours without the variant of interest), as well any normal samples provided. Increasing the number of the set of control samples, while computationally more expensive, increases the statistical robustness of the results obtained.

**Figure 1.**
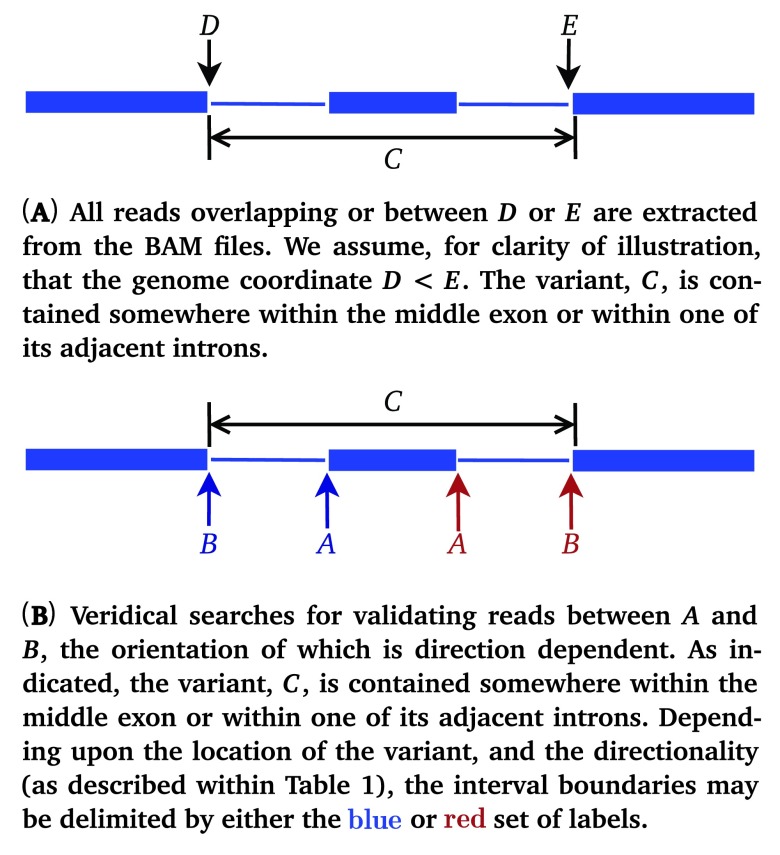
Diagram portraying the definitions used within Veridical to specify genic variant position and read coordinates. We employ the same conventions as IGV ^14^. Blue lines denote genes, wherein thick lines represent exons and thin lines represent introns.

For each variant, Veridical directly analyzes sequence reads aligned to the exons and introns that are predicted to be affected by the genomic variant. We elected to avoid indirect measures of exon skipping, such as loss of heterozygosity in the transcript, because of the possibility of confusion with other molecular etiologies (i.e. deletion or gene conversion), unrelated to the splicing mutations. The nearest natural site is found using the exome annotation file provided, based upon the directionality of the variant, as defined within Table 1. The genomic coordinates of the neighboring exon boundaries are then found and the program proceeds, iterating over all known transcript variants for the given gene. A diagram of this procedure is provided in Figure 1. The variant location, *C*, is specifically referring to the variant itself. *J _C_* refers to the variant-induced location of the predicted mRNA splice site, which is often proximate to, but distinct from the coordinate of the actual genomic mutation itself.

**Table 1.** Definitions used within Veridical to determine the direction in which reads are checked. *A* and *B* represent natural site positions, defined in Figure 1(B).

Pertinent Splice Site			
*A*	*B*	Strand	Direction
Exonic	Donor *^α^*	+	→
Exonic	Donor *^α^*	-	←
Intronic	Acceptor *^β^*	+	←
Intronic	Acceptor *^β^*	-	→

*^α^* – 5′ splice site *^β^* – 3′ splice site

**Figure 2.**
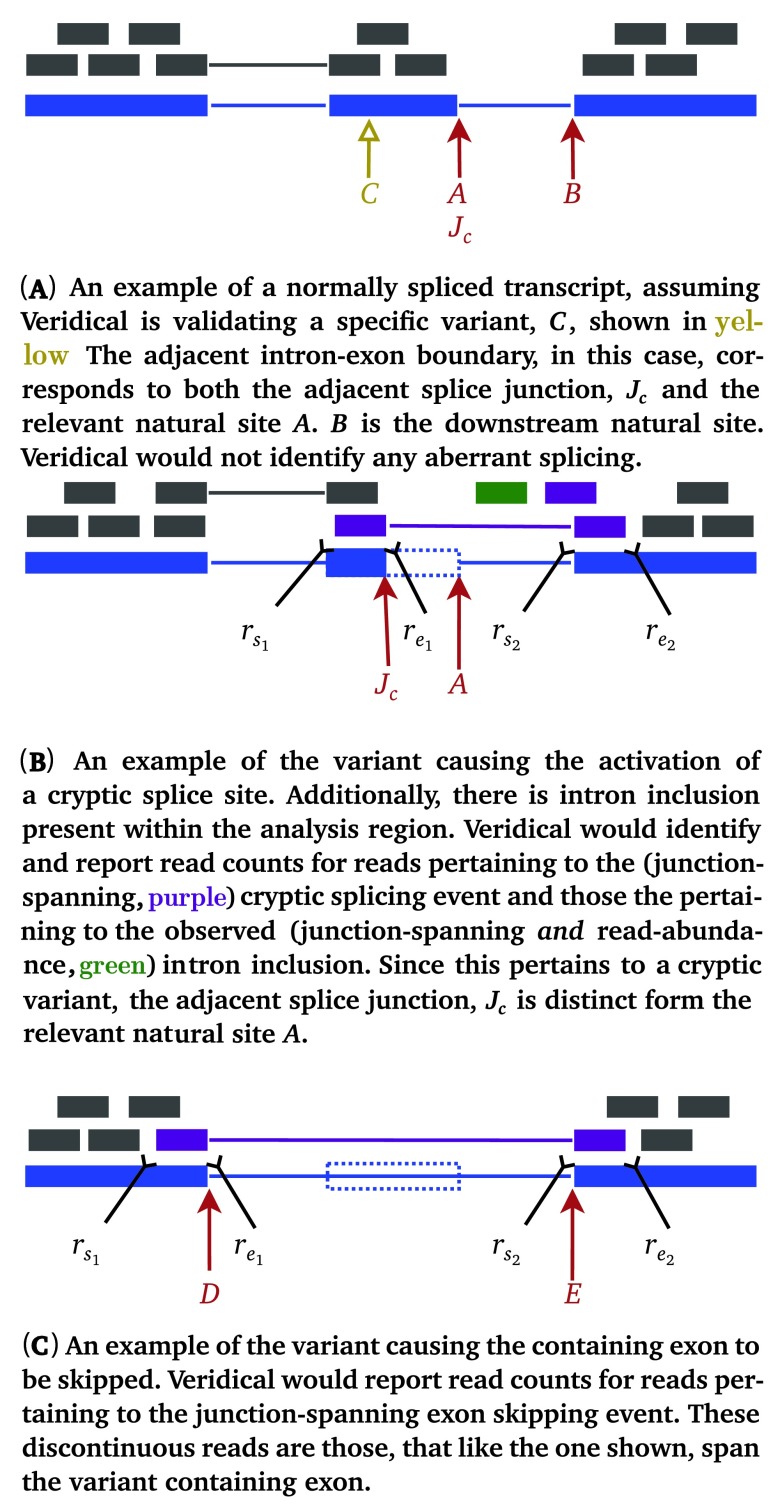
Illustrative examples of aberrant splicing detection. Grey lines denote reads, wherein thick lines denote a read mapping to genomic sequence and thin lines represent connecting segments of reads split across spliced-in regions (i.e. exons or included introns). Dotted blue rectangles denote portions of genes which are spliced out in a mutant transcript, but are otherwise present in a normal transcript. Mutant reads are purple if they are junction-spanning and green if they are read-abundance based. Start and end coordinates of reads with two portions are denoted by ( *r*_*s*_1__, *r*_*e*_1__) and ( *r*_*s*_2__, *r*_*e*_2__), while coordinates of those with only a single portion are denoted by ( *r _s_*, *r _e_*). Refer to the caption of Figure 1 for additional graphical element descriptions.

**Figure 3.**
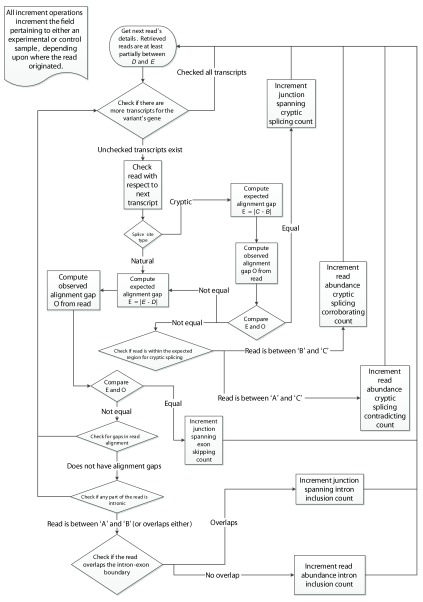
The algorithm employed by Veridical to validate variants. Refer to Table 1 for definitions concerning direction and Figure 1 for variable depictions. B is defined as follows: *B* ( *B* site left (←) of *A* ⇒ *B* := *D*. *B* site right (→) of *A* ⇒ *B* := *E*.

The program uses the BamTools API ^15^ to iterate over all of the reads within a given genomic region across experimental and control samples. Individual reads are then assessed for their corroborating value towards the analysis of the variant being processed, as outlined in the flowchart in Figure 3. Validating reads are based on whether they alter either the location of the splice junction (i.e. junction-spanning) or the abundance of the transcript, particularly in intronic regions (i.e. read-abundance). Junction-spanning reads contain DNA sequences from two adjacent exons or are reads that extend into the intron ( Equation 1(e)). These reads directly show whether the intronic sequence is removed or retained by the spliceosome, respectively. Read-abundance validated reads are based upon sequences predicted to be found in the mutated transcript in comparison with sequences that are expected to be excised from the mature transcript in the absence of a mutation ( Equation 1(f)). Both types of reads can be used to validate cryptic splicing, exon skipping, or intron inclusion. A read is said to corroborate cryptic splicing if and only if the variant under consideration is expected to activate cryptic splicing. Junction-spanning, cryptic splicing reads are those in which a read is exactly split from the cryptic splice site to the adjacent exon junction ( Equation 1(a)). For read-abundance cryptic splicing, we define the concept of a read fraction, which is the ratio of the number of reads corroborating the cryptically spliced isoform and the number of reads that do not support the use of the cryptic splice site (i.e. non-cryptic corroborating) in the same genomic region of a sample. Cryptic corroborating reads are those which occur within the expected region where cryptic splicing occurs (i.e. spliced-in regions). This region is bounded by the variant splice site location and the adjacent (direction dependent) splice junction ( Equation 1(a)). Non-cryptic corroborating reads, which we also term “anti-cryptic” reads, are those that do not lie within this region, but would still be retained within the portion that would be excised, had cryptic splicing occurred ( Equation 1(b)). To identify instances of exon skipping, Veridical only employs junction-spanning reads. A read is considered to corroborate exon skipping if the connecting read segments are split such that it connects two exon boundaries, skipping an exon in between ( Equation 1(c)). A read is considered to corroborate intron inclusion when the read is continuous and either overlaps with the intron-exon boundary (and is then said to be junction-spanning) or if the read is within an intron (and is then said to be based upon read-abundance). We only consider an intron inclusion read to be junction spanning if it spans the relevant splice junction, *A*. Equation 1(d) formalizes this concept. We occasionally use the term “total intron inclusion” to denote that any such count of intron inclusion reads includes both those containing and not containing the mutation itself. Graphical examples of some of these validation events, with a defined variant location, are provided in Figure 2.

We proceed to formalize the above descriptions as follows. A given read is denoted by *r*, with start and end coordinates ( *r _s_*, *r _e_*), if the read is continuous, or otherwise, with start and end coordinate pairs, ( *r*_*s*_1__, *r*_*e*_1__) and ( *r*_*s*_2__, *r*_*e*_2__) as diagrammed within Figure 2. Let *ℓ* be the length of the read. The set *ζ* denotes the totality of validating reads. The criterion for *r ∈ ζ* is detailed below. It is important to note that validating reads are necessary but not sufficient to validate a variant. Sufficiency is achieved only if the number of validating reads is statistically significant relative to those present in control samples. *ζ* itself is partitioned into three sets: *ζ _c_*, *ζ _e_*, and *ζ _i_* for evidence of cryptic splicing, exon skipping, and intron inclusion, respectively. We allow partitions to be empty. Let *J _C_* denote the adjacent splice junction, and let *B* denote the downstream natural site, as defined by Figure 2 and Table 1. Without loss of generality, we consider only the red (i.e. direction is right) set of labels within Figure 1(B), as further typified by Figure 2. Then the (splice consequence) partitions of *ζ* are given by:

*r* ∈ *ζ* *_c_* ⇔ variant is cryptic ∧ $(r_{s_{2}}\;–\;r_{e_{1}}$ = *B* – *J _C_* ∨ ( *r _s_* > *J _C_* ∧ *r _e_* < *A*))        (1a)

*r* ∉ *ζ* *_c_* ∧ variant is cryptic ∧ ¬ ( $r_{s_{2}}\;–\;r_{e_{1}}$ = *B* – *J _C_*) ⇒ *r* ∈ anti-cryptic        (1b)

*r* ∈ *ζ* *_e_* ⇔ $\left(r_{e_{1}}=D\wedge \;r_{s_{2}}=E\right)$        (1c)

*r* ∈ *ζ* *_i_* ⇔ ( *A*∈ [ *r _s_*, *r _e_*]) ∨ (( *A* ∉ [ *r _s_*, *r _e_*]) ∧ *r _s_* > *A* – *ℓ* ∧ *r _e_* < *B* ∧ ¬ ( *A* ∈ [ *r _s_*, *r _e_*]))        (1d)

We separately partition *ζ* by its evidence type, the set of junction-spanning reads, *δ* and read-abundance reads, *α*:

*r* ∈ *δ* ⇔ ( *A* ∈ [ *r _s_*, *r _e_*]) ∨ ( *r* ∈ *ζ* *_c_* ∧ $r_{s_{2}}\;–\;r_{e_{1}}$ = *B* – *J _C_*)        (1e)

*r* ∈ α ⇔ *r* ∉ *δ*        (1f)

Once all validating reads are tallied for both the experimental and control samples, a p-value is computed. This is determined by computing a z-score upon Yeo-Johnson (YJ) ^16^ transformed data. This transformation, shown in Equation 2, ensures that the data is sufficiently normally distributed to be amenable to parametric testing.

$$\psi \;(x,\lambda )=\left\{ \begin{array}{l} \frac{{\left(x+1\right)}^{\lambda }-1}{\lambda }\;\;\;\;\;\mathrm{if}x\geq \text{0}\wedge \lambda \neq \text{0}\\ \log(x+1)\;\;\;\;\;\;\mathrm{if}x\geq \text{0}\wedge \lambda =\text{0}\\ -\frac{{\left(-x+1\right)}^{2-\lambda }-1}{2-\lambda }\;\;\mathrm{if}x<\text{0}\wedge \lambda \neq 2\\ -\log(-x+1)\;\;\;\;\mathrm{if}x<\text{0}\wedge \lambda =2 \end{array}\right.\;\;\;\;(2)$$

**Figure S1.**
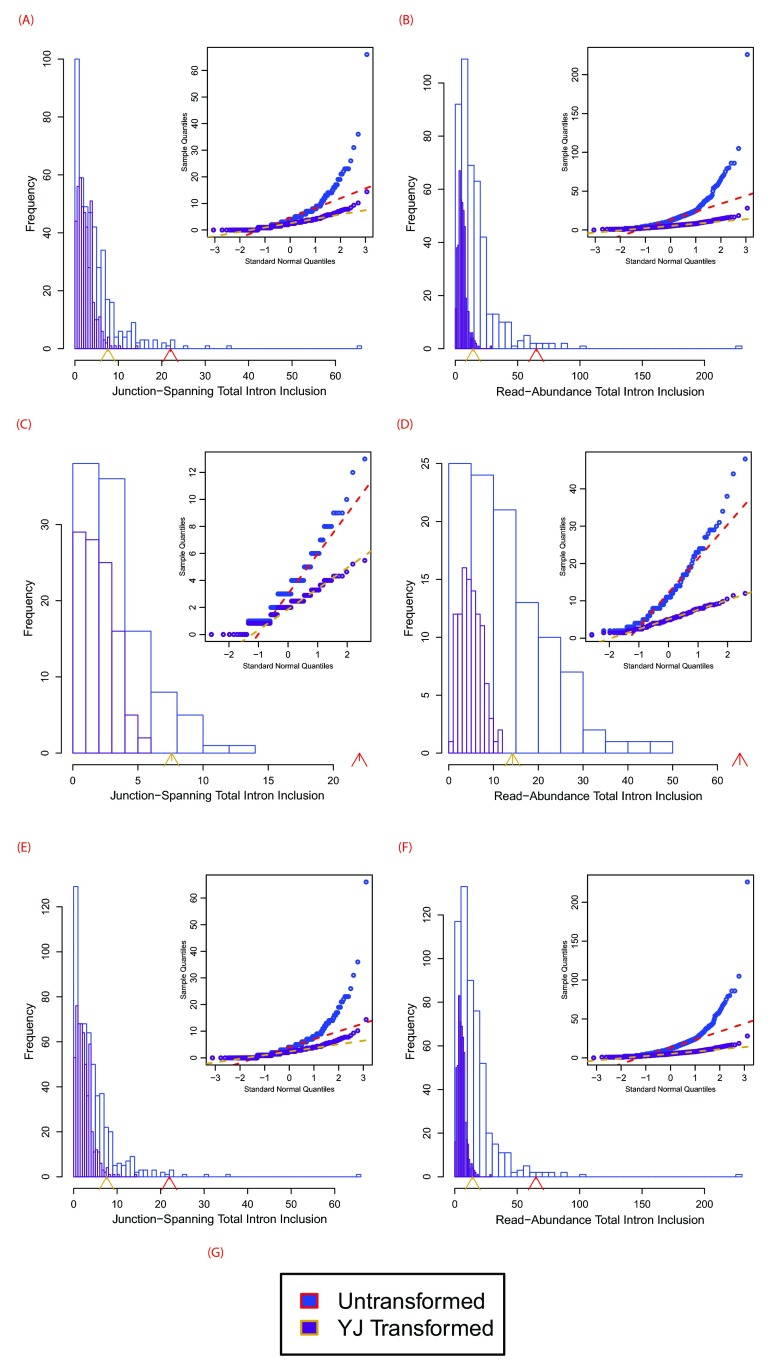
Histogram and embedded Q-Q plots portraying the difference between untransformed and Yeo-Johnson (YJ) transformed data. The plots depict intron inclusion for the inactivating mutation (chr12:83359523G>A) within *TMTC2*, as shown in Figures 6(B) and 6(C). The arrowheads denote the number of reads in the variant-containing file, which is, in all cases, more than observed in the control samples ( *p* < 0.01). The figure legend for all panels is provided in ( **G**), which shows that blue and red plot elements correspond to untransformed data, while yellow and purple correspond to YJ transformed elements. Dotted lines in the Q-Q plots are lines passing through the first and third quantiles for a normal reference distribution. ( **A**), ( **C**), and ( **E**) show junction-spanning based reads, while ( **B**), ( **D**), and ( **F**) show read-abundance-based reads. ( **A**)–( **B**) depict tumour sample distributions, ( **B**)–( **C**) depict normal sample distributions, and ( **E**)–( **F**) depict combined tumour and normal sample distributions. This figure is demonstrative of the general trend we have observed. Only data from normal samples resemble a Gaussian distribution and the YJ transformation greatly improves the Gaussian nature of all distributions.

The transform is similar to the Box-Cox power transformation, but obviates the requirement of inputting strictly positive values and has more desirable statistical properties. Furthermore, this transformation allowed us to avoid the use of non-parametric testing, which has its own pitfalls regarding assumptions of the underlying data distribution ^17^. We selected *λ* = $\frac{1}{2}$, because Veridical’s untransformed output is skewed left, due to their being, in general, less validating reads in control samples and the fact that there are, by design, vastly more control samples than experimental samples. We found that this value for *λ* generally made the distribution much more normal. A comparison of the distributions of untransformed and transformed data is provided in Figure S1. We were not concerned about small departures from normality as a z-test with a large number of samples is robust to such deviations ^18^.

Thus, we can compute the p-value of the pairwise unions of the two sets of partitions of *ζ*, except the irrelevant *ζ _e_* ∪ *α* = Ø. We only provide p-values for these pairwise unions and do not attempt to provide p-values for the partitions for the different consequences of the mutations on splicing. While such values would be useful, we do not currently have a robust means to compute them. Our previous work provides guidance on interpretation of splicing mutation outcomes ^3– 5, 10^. Thus for *ζ _x_* ∈ { *ζ _c_*, *ζ _e_*, *ζ _i_*}, let Φ *_Z_* ( *z*) represent the cumulative distribution function of the one-sided (right-tailed — i.e. *P*[ *X > x*]) standard normal distribution. Let *N* represent the total number of samples and let V represent the set of all *ζ _x_* validations, across all samples. Then:

$$\begin{array}{l} \mu =\frac{\sum_{j=1}^{N}V_{j}}{N}\;\;\;\;\sigma =\sqrt{\frac{1}{N}\sum_{j=1}^{N}(V_{j}-\bar{V}){}_{}^{2}}\\ z=\frac{|\zeta_{x}|-\mu }{\sigma }\;\;\;p=\text{Φ}(\psi (z,\frac{1}{2})) \end{array}$$

The program outputs two tables, along with summaries thereof. The first table lists all validated read counts across all categories for experimental samples, while the second table does the same for the control samples. P-values are shown in parentheses within the experimental table, which refer to the column-dependent (i.e. the read type is given in the column header) p-value for that read type with respect to that same read type in control samples. The program produces three files: a log file containing all details regarding validated variants, an output file with the programs progress reports and summaries, and a filtered validated variant file. The filtered file contains all validated variants of statistical significance (set as *p* < 0.05, by default), defined as variants with one or more validating reads achieving statistical significance in a strongly corroborating read type. These categories are limited to all junction-spanning based splicing consequences and read-abundance total intron inclusion. For example, a cryptic variant for which *p* = 0.04 in the junction-spanning cryptic column would meet this criteria, assuming the default significance threshold.

The p-values given by Veridical are more robust when the program is provided with a large number of samples. The minimum sample size is dependent upon the desired power, *α* value, and the effect size ( *ES*). The minimum samples size could be computed as follows: $N=\left\lceil \frac{\sigma^{2}z^{2}}{ES^{2}}\right\rceil$. For *α* = 0.05 and *β* = 0.2 (for a power of 0.8): *z* = 2.4865 for the one-tailed test. for the one-tailed test. Then, $N=\left\lceil \frac{\sigma^{2}2.4865^{2}}{ES^{2}}\right\rceil$. Ideally, Veridical could be run with a trial number of samples.

Then, one would compute effect sizes from Veridical’s output. The standard deviation in the above formula could also be estimated from one’s data, although it should be transformed using Yeo-Johnson (such as via an appropriate R package) before computing this estimation.

We elected to use RefSeq ^19^ genes for the exome annotation, as opposed to, the more permissive exome annotation sets, UCSC Known Genes ^20^ or Ensembl ^21^. The large number of transcript variants within Ensembl, in particular, caused many spurious intron inclusion validation events. This occurred because reads were found to be intronic in many cases, when in actuality they were exonic with respect to the more common transcript variant. In addition, the inclusion of the large number of rare transcripts in Ensembl significantly increased program run-time and made validation events much more challenging to interpret unequivocally. The use of RefSeq, which is a conservative annotation of the human exome, resolves these issues. It is possible that some subset of unknown or Ensemble annotated intronic transcripts could be sufficiently prevalent to merit inclusion in our analysis. We do not attempt to perform the difficult task of deciding which of these transcripts would be worth using. Indeed, the task of confirming and annotating of such transcripts is already done by the more conservative annotation we employ.

We also provide an R program ^22^ which produces publication quality histograms displaying embedded Q-Q plots and p-values, to evaluate for normality of the read distribution and statistical significance, respectively. The R program performs the YJ transformation as implemented in the **car** package ^23^. The histograms generated by the program use the Freedman-Draconis ^24^ rule for break determination, and the Q-Q plots use algorithm Type 8 for their quantile function, as recommended by Hyndman and Fan ^25^. This program is embedded within a Perl script, for better integration into our workflow. Lastly, a Perl program was implemented to automatically retrieve and correctly format an exome annotation file from the UCSC database ^20^ for use in Veridical. All data use hg19/GRCh37, however when new versions of the genome become available, this program can be used to update the annotation file.

## Results

Veridical validates predicted mRNA splicing mutations using high-throughput RNA sequencing data. We demonstrate how Veridical and its associated R program are used to validate predicted splicing mutations in somatic breast cancer. Each example depicts a particular variant-induced splicing consequence, analyzed by Veridical, with its corresponding significance level. The relevant primary RNA-Seq data are displayed in IGV, along with histograms and Q-Q plots showing the read distributions for each example. The source data are obtained from controlled-access breast carcinoma data from The Cancer Genome Atlas (TCGA) ^26^. Tumour-normal matched DNA sequencing data from the TCGA consortium was used to predict a set of splicing mutations, and a subset of corresponding RNA sequencing data was analyzed to confirm these predictions with Veridical. Overall, 442 tumour samples and 106 normal samples were analyzed. Briefly, all variants used as examples in this manuscript came from running the matched TCGA exome files (to which the RNA-Seq data corresponds) through SomaticSniper ^27^ and Strelka ^28^ to call somatic mutations, followed by the Shannon Human Splicing Pipeline ^10^ to find splicing mutations, which served as the input to Veridical. Details of the RNA-Seq data can be found within the supplementary methods of the TCGA paper ^26^. Accordingly, the following examples demonstrate the utility of Veridical to identify potentially pathogenic mutations from a much larger subset of predicted variants.

Input, output, and explanatory files for Veridical VeridicalOutExample.xls: contains the output for the variant within RAD54L, along with descriptions of the terms used and the output format.
all.vin: contains the full set of input variants for Veridical used in this paper.
allTumoursBAMFileList.txt: The list of BAM files for the RNASeq data of the breast tumour samples analyzed. This contains TCGA file UUIDs, followed by a slash, followed by the file names themselves.
allNormalsBAMFileList.txt: The list of BAM files for the RNASeq data of the normal samples corresponding to these tumors. This contains TCGA file UUIDs, followed by a slash, followed by the file names themselves.
all.vout: The full Veridical output produced when running Veridical with all.vin.

### Leaky Mutations

**Figure 4.**
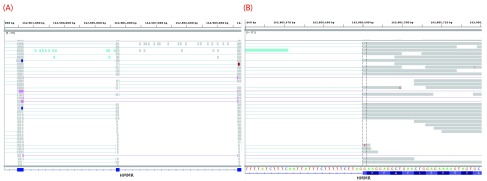
IGV images depicting a predicted leaky mutation (chr5:162905690G>T) within the natural acceptor site of exon 12 (162905689–162905806) of *HMMR*. This gene has four transcript variants and the given exon number pertains to isoforms *a* and *b* (reference sequences **NM_001142556** and **NM_012484**). RNA-Seq reads are shown in the centre panel. The bottom blue track depicts RefSeq genes, wherein each blue rectangle denotes an exon and blue connecting lines denote introns. In the middle panel, each rectangle (grey by default) denotes an aligned read, while thin lines are segments of reads split across exons. Red and blue coloured rectangles in the middle panel denote aligned reads of inserts that are larger or smaller than expected, respectively. Reads are highlighted by their splicing consequence, as follows: cryptic splicing ( green), exon skipping ( purple), junction-spanning intron inclusion ( dark green), and read-abundance intron inclusion ( cyan). ( **A**) depicts a genomic region of chromosome 5: 162902054–162909787. The variant occurs in the middle exon. Intron inclusion can be seen in this image, represented by the reads between the first and middle exon (since the direction is left, as described within Table 1). These 14 reads are read-abundance-based, since they do not span the intron-exon junction. ( **B**) depicts a closer view of the region shown in ( **A**) — 162905660–162905719. The dotted vertical black lines are centred upon the first base of the variant-containing exon. The thin lines in the middle panel that span the entire exon fragment are evidence of exon skipping. These 5 reads are split across the exon before and after the variant-containing exon, as seen in ( **A**).

**Figure 5.**
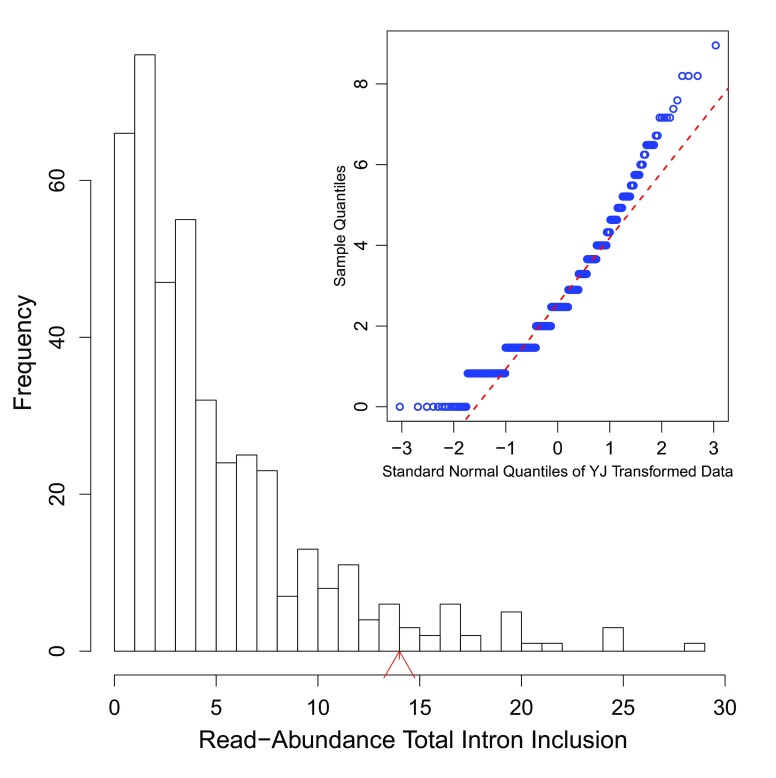
Histogram of read-abundance-based intron inclusion with embedded Q-Q plots of the predicted leaky mutation (chr5:162905690G>T) within *HMMR*, as shown in Figure 4. The arrowhead denotes the number of reads (14 in this case) in the variant-containing file, which is more than observed in the control samples ( *p* = 0.04).

Mutations that reduce, but not abolish, the spliceosome’s ability to recognize the intron/exon boundary are termed leaky ^3^. This can lead to the mis-splicing (intron inclusion and/or exon skipping) of many but not all transcripts. An example, provided in Figure 4, displays a predicted leaky mutation (chr5:162905690G>T) in the *HMMR* gene in which both junction-spanning exon skipping ( *p* < 0.01) and read-abundance-based intron inclusion ( *p* = 0.04) are observed. We predict this mutation to be leaky because its final *R _i_* exceeds 1.6 bits — the minimal individual information required to recognize a splice site and produce correctly spliced mRNA ^4^. Indeed, the natural site, while weakened by 2.16 bits, remains strong — 10.67 bits. This prediction is validated by the variant-containing sample’s RNA-Seq data ( Figure 4), in which both exon skipping (5 reads) and intron inclusion (14 reads, 12 of which are shown, versus an average of 4.051 such reads per control sample) are observed, along with 70 reads portraying wild-type splicing. Only a single normally spliced read contains the G *→*T mutation. These results are consistent with an imbalance of expression of the two alleles, as expected for a leaky variant. Figure 5 shows that for the distribution of read-abundance-based intron inclusion is marginally statistically significant ( *p* = 0.04).

### Inactivating Mutations

**Figure 6.**
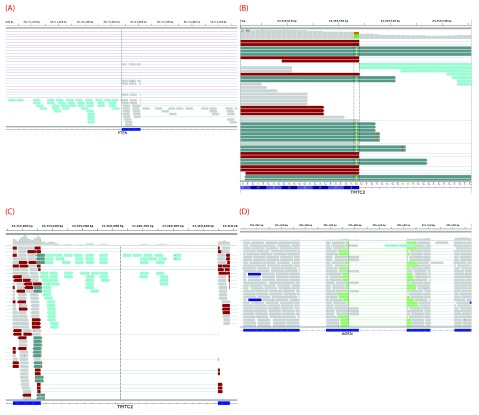
( **A**) depicts an inactivating mutation (chr10:89711873A>G) within the natural acceptor site of exon 6 (89711874–89712016) of *PTEN*. The dotted vertical black line denotes the location of the relevant splice site. The region displayed is 89711004–89712744 on chromosome 10. Many of the 32 exon skipping reads are evident, typified by the thin lines in the middle panel that span the entire exon. There is also a substantial amount of read-abundance-based intron inclusion, shown by the reads to the left of the dotted vertical line. Exon skipping was statistically significant ( *p* < 0.01), while read-abundance-based intron inclusion was not ( *p* = 0.53). Panels ( **B**) and ( **C**) depict an inactivating mutation (chr12:83359523G>A) within the natural donor site of exon 6 (83359338–83359523) of *TMTC2*. ( **B**) depicts a closer view (83359501–83359544) of the region shown in ( **C**) and only shows exon 6. Some of the 22 junction-spanning intron inclusion reads can be seen. In this case, all of these reads contain the mutation, shown by the green adenine base in each read, between the two vertical dotted lines. ( **C**) depicts a genomic region of chromosome 12: 83359221–83360885, *TMTC2* exons 6–7. The variant occurs in the left exon. 65 read-abundance-based intron inclusion can be seen in this image, represented by the reads between the two exons. Panel ( **D**) depicts a mutation (chr1:985377C>T) causing a cryptic donor to be activated within exon 27 (the second from left, 985282–985417) of *AGRN*. The region displayed is 984876–985876 on chromosome 1 (exons 26–29 are visible). Some of the 34 cryptic (junction-spanning) reads are portrayed. The dotted black vertical line denotes the cryptic splice site, at which cryptic reads end. The read-abundance-based intron inclusion, of which two reads are visible, was not statistically significant ( *p* = 0.68). Refer to the caption of Figure 4 for IGV graphical element descriptions.

Variants that inactivate splice sites have negative final *Ri* values ^3^ with only rare exceptions ^4^, indicating that splice site recognition is essentially abolished in these cases. We present the analysis of two inactivating mutations within the *PTEN* and *TMTC2* genes from different tumour exomes, namely: chr10:89711873A>G and chr12:83359523G>A, respectively. The *PTEN* variant displays junction-spanning exon skipping events ( *p* < 0.01), while the *TMTC2* gene portrays both junction-spanning and read-abundance-based intron inclusion (both splicing consequences with *p* < 0.01). In addition, all intron inclusion reads in the experimental sample contain the mutation itself, while only one such read exists across all control samples analyzed ( *p* < 0.01). The *PTEN* variant contains numerous exon skipping reads (32 versus an average of 2.466 such reads per control sample). The *TMTC2* variant contains many junction-spanning intron inclusion reads with the G *→*A mutation (all of its junction-spanning intron inclusion reads: 22 versus an average of 0.002 such reads per control sample). IGV screenshots for these variants are provided within Figure 6. This figure also shows an example of junction-spanning cryptic splice site activated by the mutation (chr1:985377C>T) within the *AGRN* gene. The concordance between the splicing outcomes generated by these mutations and the Veridical results indicates that the proposed method detects both mutations that inactivate splice sites and cryptic splice site activation.

### Cryptic Mutations

**Figure 7.**
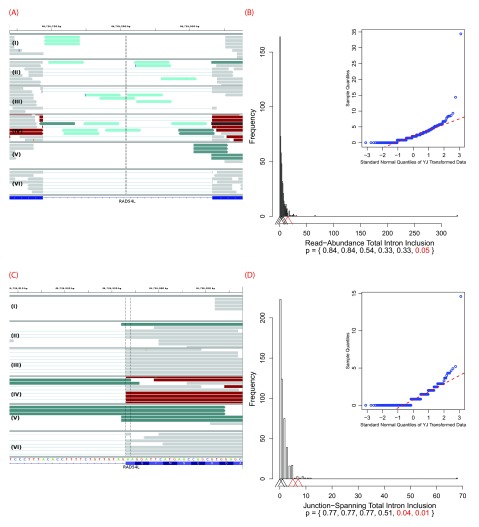
IGV images and their corresponding histograms with embedded Q-Q plots depicting all six variant-containing files with a mutation (chr1:46726876G>T) which, in some cases, causes a cryptic donor to be activated within the intron between exons 7 and 8 of *RAD54L*. This results in the extension of the downstream natural donor (the 5′ end of exon 8). This gene has two transcript variants and the given exon numbers pertain to isoform *a* (reference sequence **NM_003579**). Only samples IV and V have statistically significant intron inclusion relative to controls. read-abundance-based intron inclusion can be seen in ( **A**), between the two exons. The region displayed is on chromosome 1: 46726639–46726976. ( **B**) depicts the corresponding histogram for the 15 read-abundance-based intron inclusion reads ( *p* = 0.05) that are present in sample IV. The intron-exon boundary on the right is the downstream natural donor. ( **C**) typifies some of the 13 junction-spanning intron inclusion reads that are a direct result of the intronic cryptic site’s activation. In these instances, reads extending past the intron-exon boundary are being spliced at the cryptic site, instead of the natural donor. In particular, samples IV and V both have a statistically significant numbers of such reads, 7 ( *p* = 0.01) and 5 ( *p* = 0.04), respectively. This is further typified by the corresponding histogram in ( **D**). ( **C**) focuses upon exon 8 from ( **A**) and displays the genomic positions 46726908–46726957. Refer to the caption of Figure 4 for IGV graphical element descriptions. In the histograms, arrowheads denote numbers of reads in the variant-containing files. The bottom of the plots provide p-values for each respective arrowhead. Statistically significant p-values and their corresponding arrowheads are denoted in red.

Recurrent genetic mutations in some oncogenes have been reported among tumours within the same, or different, tissues of origin. Common recurrent mutations present in multiple abnormal samples are recognized by Veridical. This avoids including a variant-containing sample among the control group, and outputs the results of all of the variant-containing samples. A relevant example is shown in Figure 7. The mutation (chr1:46726876G>T) causes activation of a cryptic splice site within *RAD54L* in multiple tumours. Upon computation of the p-values for each of the variant-containing tumours, relative to all non-variant containing tumours and normal controls, not all variant-containing tumours displayed splicing abnormalities at statistically significant levels. Of the six variant-containing tumours, two had significant levels of junction-spanning intron inclusion, and one showed statistically significant read-abundance-based intron inclusion. Details for all of the aforementioned variants, including a summary of read counts pertaining to each relevant splicing consequence, for experimental versus control samples, are provided in Table 2.

**Table 2.** Examples of variants validated by Veridical and their selected read types. Header abbreviations Chr, *C _v_*, *C _s_*, *#*, SC, and ET, denote chromosome, variant coordinate, splice site coordinate, sample number (where applicable), splicing consequence, and evidence type, respectively. Headers containing R with some subscript denote numbers of validated reads for the specified variant’s splicing consequence(s) and evidence type(s). *R _E_* denotes reads within variant-containing tumour samples. *R _T_* and *R _N_* denote control samples, for tumours and normal cells, respectively. *R _μ_* is the per sample mean of *R _T_* and *R _N_*. Splicing consequences: CS denotes cryptic splicing, ES denotes exon skipping, and II denotes intron inclusion. Evidence types: JS denotes junction-spanning and RA denotes read-abundance.

Gene	Chr	*C _v_*	*C _s_*	Variant	Type	Initial *R _i_*	Final *R _i_*	Δ *R _i_*	#	SC	ET	p-value	*R _E_*	*R _T_*	*R _N_*	*R _μ_*	Figure
*HMMR*	chr5	162905690	162905689	G/T	Leaky	12.83	10.67	-2.16		ES	JS	< 0.01	5	11	0	0.020	4, 5
										II	RA	0.04	14	2133	103	4.051	
*PTEN*	chr10	89711873	89711874	A/G	Inactivating	12.09	-2.62	-14.71		ES	JS	< 0.01	32	975	386	2.466	6(A)
*TMTC2*	chr12	83359523	83359524	G/A	Inactivating	1.74	-1.27	-3.01		II	JS	< 0.01	22	2241	383	4.754	6(B)
										II	JSwM	< 0.01	22	0	1	0.002	
										II	RA	< 0.01	65	7293	1395	15.739	6(C)
*AGRN*	chr1	985377	985376	C/T	Cryptic	-2.24	4.79	7.03		CS	JS	< 0.01	34	97	23	0.217	6(D)
*RAD54L*	chr1	46726876	46726895	G/T	Cryptic	13.4	14.84	1.44	I	II	JS	N/A	0	645	58	1.274	7
										II	RA	0.54	3	2171	290	4.458	
									II	II	JS	0.51	1	645	58	1.274	
										II	RA	0.33	6	2171	290	4.458	
									III	II	JS	N/A	0	645	58	1.274	
										II	RA	0.33	6	2171	290	4.458	
									IV	II	JS	0.01	7	645	58	1.274	
										II	RA	0.05	15	2171	290	4.458	
									V	II	JS	0.04	5	645	58	1.274	
										II	RA	N/A	0	2171	290	4.458	
									VI	II	JS	N/A	0	645	58	1.274	
										II	RA	N/A	0	2171	290	4.458	

### Performance

**Figure 8.**
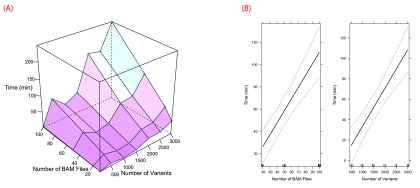
Profiling data for Veridical runtime. Tests were conducted upon an Intel Xeon @ 2.27 GHz. Visualizations were generated with R ^22^ using Lattice ^30^ and Effects ^31^. A surface plot of time vs. numbers of BAM files and variants is provided in ( **A**). Effect plots are given in ( **B**) and demonstrate the effects of the numbers of BAM files and variants upon runtime. The effect plots were generated using a linear regression model ( *R* ^2^ = 0.7525).

The performance of the software is affected by the number of predicted splicing mutations, the number of abnormal samples containing mutations and control samples and the corresponding RNA-Seq data for each type of sample. Veridical has the ability to analyze approximately 3000 variants in approximately 4 hours, assuming an input of 100 BAM files of RNA-Seq data. The relationship between time and numbers of BAM files and variants are plotted in Figure 8 for a 2.27 GHz processor. Veridical uses memory in linear proportion to the number and size of the input BAM files. In our tests, using RNA-Seq BAM files with an average size of approximately 6 GB, Veridical used approximately 0.7 GB for ten files to 1 GB for 100 files.

## Discussion

We have implemented Veridical, a software program that automates confirmation of mRNA splicing mutations by comparing sequence read-mapped expression data from samples containing variants that are predicted to cause defective splicing with control samples lacking these mutations. The program objectively evaluates each mutation with statistical tests that determine the likelihood of and exclude normal splicing. To our knowledge, no other software currently validates splicing mutations with RNA-Seq data on a genome-wide scale, although many applications can accurately detect conventional alternative splice isoforms (i.e. ^29^). Veridical is intended for use with large data sets derived from many samples, each containing several hundred variants that have been previously prioritized as likely splicing mutations, regardless of how the candidate mutations are selected. It is not practical to analyze all variants present in an exome or genome, rather only a filtered subset, due to the extensive computations required for statistical validation. As such, Veridical is a key component of an end-to-end, hypothesis-based, splicing mutation analysis framework that also includes the Shannon splicing mutation pipeline ^10^ and the Automated Splice Site Analysis and Exon Definition server ^5^. There is a trade-off between lengthy run-times and statistical robustness of Veridical, especially when there are either a large number of variants or a large number of RNA-Seq files. As with most statistical methods, those employed here are not amenable to small sample sets, but become quite powerful when a large number of controls are employed. In order to ensure that mutations can be validated, we recommend an excess of control transcriptome data relative to those from samples containing mutations (> 5 : 1), guided by the power analysis described in Methods. We *do not* recommend the use of a single nor a few control samples to corroborate a putative mutation. Not surprisingly, we have found that junction-spanning reads have the greatest value for corroborating cryptic splicing and exon skipping. Even a single such read is almost always sufficient to merit the validation of a variant, provided that sufficient control samples are used. For intron inclusion, both junction-spanning and read-abundance-based reads are useful and a variant can readily be validated with either, provided that the variant-containing experimental sample(s) show a statistically significant increase in the presence of either form of intron inclusion corroborating reads.

Veridical is able to automatically process variants from multiple different experimental samples, and can group the variant information if any given mutation is present in more than one sample. The use of a large sample size allows for robust statistical analyses to be performed, which aid significantly in the interpretation of results. The main utility of Veridical is to filter through large data sets of predicted splicing mutations to prioritize the variants. This helps to predict which variants will have a deleterious effect upon the protein product. Veridical is able to avoid reporting splicing changes that are naturally occurring through checking all variant-containing and non-containing control samples for the predicted splicing consequence. In addition, running multiple tumour samples at once allows for manual inspection to discover samples that contained the alternative splicing pattern, and consequently, permits the identification of DNA mutations in the same location which went undetected during genome sequencing.

The statistical power of Veridical is dependent upon the quality of the RNA-Seq data used to validate putative variants. In particular, a lack of sufficient coverage at a particular locus will cause Veridical to be unable to report any significant results. A coverage of at least 20 reads should be sufficient. This estimate is based upon alternative splicing analyses in which this threshold was found to imply concordance with microarray and RT-PCR measurements ^32– 35^. There are many potential legitimate reasons why a mutation may not be validated: (a) A lack of gene expression in the variant containing tumour sample, (b) nonsense-mediated decay may result in a loss of expression of the entire transcript, (c) the gene itself may have multiple paralogs and reads may not be unambiguously mapped, (d) other non-splicing mutations could account for a loss of expression, and (e) confounding natural alternative splicing isoforms may result in a loss of statistical significance during read mapping of the control samples. The prevalence of loci with insufficient data is dependent upon the coverage of the sequencing technology used. As sequencing technologies improve, the proportion of validated mutations is expected to increase. Such an increase would mirror that observed for the prevalence of alternative splicing events ^36^. In addition, mutated splicing factors can disrupt splicing fidelity and exon definition ^37^. This effect could decrease Veridical’s ability to validate splicing mutations affected by a disruption of the definition of the pertinent exon. Veridical does not currently form any equivalence between distinct variants affecting the same splice site. Such variants will be analyzed independently. Veridical is intended to be used with RNA-Seq data that not only corresponds to matched DNA-Seq data, but also only for sets of samples with comparable sequencing protocols, since the non-normalized comparisons performed rely upon the evening out of batch effects, due to a substantial number of control samples. It is important to note that acceptance of the null hypothesis, due to an absence of evidence required to disprove it, does not imply that the underlying prediction of a mutation at a particular locus is incorrect, but merely that the current empirical methods employed were insufficient to corroborate it.

“Validate,” in the present context, refers to the condition where sufficient statistical evidence has been marshaled in support of a variant. However, the threshold for significance can vary so these analyses can also be thought of as strongly corroborating variants. Recent studies in Bayesian statistics have suggested that a p-value threshold of 0.05 does not correspond to strong support of the alternative hypothesis. Accordingly, Johnson ^38^ recommends the use of tests at the 0.005 or 0.001 level of significance.

We consider alternative splicing to be a different problem. Veridical does not aim to identify putatively pathogenic variants, but rather, *to confirm* existing *in silico* predictions thereof. We do infer exon skipping events (i.e. alternative splicing) *de novo*, but only to catalog dysregulated splicing “phenotypes” due to genomic sequence variants. This is not the first study to use a large control dataset. Indeed the Variant Annotation, Analysis & Search Tool (VAAST) ^39^ does this to search for disease-causing (non-splicing) variants and the Multivariate Analysis of Transcript Splicing (MATS) ^29^ tool (among others) can be used for the discovery of alternative splicing events. However, in our case, in most instances the distribution of reads in a single sample is compared to the distributions of reads in the control set, as opposed to a likelihood framework-based approach. We are suggesting that our approach be coupled to existing approaches to act as an *a posteriori*, hypothesis-driven, check on the veridicality of specific variants.

While there is considerable prior evidence for splicing mutations that alter natural and cryptic splice site recognition, we were somewhat surprised at the apparent high frequency of statistically significant intron inclusion revealed by Veridical. In fact, evidence indicates that a significant portion of the genome is transcribed ^36^, and it is estimated that 95% of known genes are alternatively spliced ^32^. Defective mRNA splicing can lead to multiple alternative transcripts including those with retained introns, cassette exons, alternate promoters/terminators, extended or truncated exons, and reduced exons ^40^. In breast cancer, exon skipping and intron retention were observed to be the most common form of alternative splicing in triple negative, non-triple negative, and HER2 positive breast cancer ^41^. In normal tissue, intron retention and exon skipping has been predicted to affect 2572 exons in 2127 genes and 50 633 exons in 12 797 genes, respectively ^42^. In addition, previous studies suggest that the order of intron removal can influence the final mRNA transcript composition of exons and introns ^43^. Intron inclusion observed in normal tissue may result from those introns that are removed from the transcript at the end of mRNA splicing. Given that these splicing events are relatively common in normal tissues, it becomes all the more important to distinguish expression patterns that are clearly due to the effects of splicing mutations — one of the guiding principles of the Veridical method.

Veridical is an important analytical resource for unsupervised, thorough validation of splicing mutations through the use of companion RNA-Seq data from the same samples. The approach will be broadly applicable for many types of genetic abnormalities, and should reveal numerous, previously unrecognized, mRNA splicing mutations in exome and complete genome sequences.

## Data availability

figshare: Input, output, and explanatory files for Veridical, http://dx.doi.org/10.6084/m9.figshare.894971 ^44^.

## Supplementary materials

### Veridical variant input format

This input format most easily accepts formatted output from the Shannon Pipeline. In particular, all variants of interest should be concatenated into a single file. Once a, tab-delimited, concatenated file has been generated, it can easily be formatted correctly by using **FilterShannonPipelineResults.pl**. All file headers must precisely match their outlined schema. One can also manually ensure the following: the header line has no quotation marks or special characters, empty columns have been replaced by a period (.) and each variant line contains only a single gene (comma-delimited gene lists must be split such that there is only one gene per line). If one wishes Veridical to consider variants pertaining to more than one experimental sample, a comma-delimited list of experimental samples, in the form of BAM file names, must be provided as the **key** column. The **key** column must always contain at least one file name that is present as the base name of one of the files listed in the BAM file list that must be passed to Veridical.

Alternatively, one can prepare the input format as follows. The header must contain at least the following, case-insensitive, values to which the file’s columns must adhere to: chromosome, splice&coordinate, strand, type, gene, location, location_type, heterozygosity, variant, input, key. The column headers need only contain the given text (i.e. a column labeled **gene_name** would be sufficient to satisfy the above requirement for a “gene” column). Column headers with ampersands (&) denote that all words joined by this symbol must be present for that column (i.e. **Splice_site_coordinate** satisfies the “splice&coordinate” requirement). The order of the columns is immaterial. The **input** column can contain any identifier for the variant and need not be unique. The **location** column specifies if the site is natural or cryptic. For Veridical, all that matters is that cryptic variants contain the word “ **cryptic**” as part of their value in this column and that non-cryptic variants do not. The **location_type** column is only used for cryptic variants and specifies if the variant is intronic or exonic. It is not currently used by the program. This column must be present but can always be set to null (i.e).

A few rows from a sample variant file is provided below (text wrapped for readability):

Chromosome     Splice_site_coordinate      Strand
Ri-initial Ri-final ∆Ri Type  Gene_Name  Location
Location_Type Loc._Rel._to_exon
Dist._from_nearest_nat._site
Loc._of_nearest_nat._site       Ri_of_nearest_nat
Cryptic_Ri_rel._nat. rsID  Average_heterozygosity
Variant_coordinate    Input_variant      Input_ID
RNASeqDirectory_ID RNA_Seq_BAM_ID_KEY
chr10 89711874 + 12.09 -2.62 -14.71 ACCEPTOR PTEN
NATURALSITE . . . . . . . . 89711873 A/G ID1  dir
file
chr10 89712017 + 5.18   -1.85  -7.03  DONOR  PTEN
NATURALSITE . . . . . . . . 89712018 T/C ID1  dir
file
chrX   9621719 + -4.78  2.25  7.03   DONOR  TBL1X
CRYPTICSITE EXONIC . 11  9621730  2.24  GREATER .
. 9621720 C/T ID1 dir file

### Veridical exome annotation input format

This input format can be generated via **ConvertToExomeAnnotation.pl**. The file must be tab-delimited, excepting its header, which must be comma-delimited. It must have the following, case-insensitive, header columns, to which its data must adhere: transcript, chromosome, exon chr start, exon chr end, exon rank, gene. The column headers need only contain the given text (i.e. a column labeled **gene_name** would be sufficient to satisfy the above requirement for a “gene” column). The order of the columns is immaterial.

A few rows from a sample exome annotation file is provided below (text wrapped for readability):

Transcript ID,ID,ID,Chromosome Name,Strand,
Exon Chr Start,Exon Chr End,
Exon Rank in Transcript,Transcript Start,
Transcript End, Associated Gene Name
NM_213590 NM_213590   NM_213590 chr13  +  50571142
50571899 1 50571142   50592603 TRIM13
NM_213590 NM_213590   NM_213590 chr13  +  50586070
50592603 2 50571142   50592603 TRIM13
NM_198318 NM_198318   NM_198318 chr19  +  50180408
50180573 1 50180408   50191707 PRMT1

### Veridical output

If a variant contains any validating reads, Veridical outputs the variant in question, along with some summary information and a table specifying the numbers of each validating read type detected for both the experimental and control samples. Within the output of Veridical, the phrase: “Validated ( *x*) variant *n* times” means that the variant was validated mainly for splicing consequence *x* and has *n* validating reads. The variant will only appear within the ***.filtered** output file if the p-value for either junction-spanning or read-abundance-based reads for splicing consequence *x* was statistically significant (defined, by default, as: *p* < 0.05). After the variant being validated is provided, along with its primary predicted splicing consequence, the output is divided into two sections with identical contents: one for the experimental sample(s) and another for control samples. The summary enumerates the number of reads of each splicing consequence, partitioned by evidence type (junction-spanning or read-abundance-based), and by sample type (tumour or normal for control samples, and only tumour for experimental samples). A table describing the number of each read type for every file follows this summary. An example of this output, for the variant within *RAD54L*, as shown by Figure 7 and the last portion of Table 2, is provided. While Veridical outputs this as plain text, with the table in a tab-delimited format, we provide this output as an Excel document with descriptions of the meaning of each table heading, to clarify the presentation of the data. All input and output files for the five variants presented are provided. **VeridicalOutExample.xls** contains the output for the variant within *RAD54L*, along with descriptions of the terms used and the output format. **all.vin** contains the input variant file. **allTumoursBAMFileList.txt** and **allNormalsBAMFileList.txt** are the BAM file lists for tumour and normal samples, respectively. **all.vout** contains the Veridical output. The exome file can be retrieved using **ConvertToExomeAnnotation.pl**, available with the other programs at: www.veridical.org. The BAM file lists contain the TCGA file UUID, followed by a slash, followed by the file name. The RNA-Seq data itself can be downloaded from TCGA at: https://tcga-data.nci.nih.gov/tcga/.
