# Supplementary material for: Validation of predicted mRNA splicing mutations using high-throughput transcriptome data
Source: F1000Res. 2014 Apr 7;3:8. Originally published 2014 Jan 13. [Version 2] doi: 10.12688/f1000research.3-8.v2 (PMC3983938; doi:10.12688/f1000research.3-8.v2)
Supplement: Peer review history

# Peer review: Referee response for version 2

**Published:** 2014-04-08

**Authors:** Stefania Bortoluzzi

**Affiliations:** 1 Department of Biology, University of Padova, Padova, Italy

The revised version of the manuscript is improved, and it incorporates all the revisions I suggested.

I have read this submission. I believe that I have an appropriate level of expertise to confirm that it is of an acceptable scientific standard.

# Peer review: Referee response for version 1

**Published:** 2014-03-18

**Authors:** Peter Robinson

**Affiliations:** 1 Institute for Medical Genetics, Universitätsklinikum Charité, Berlin, Germany

This paper from the Rogan group presents a methodology for validation of DNA sequencing variants that alter mRNA splicing. While variants of the most conserved splice site nucleotides at the intron-exon boundary can be predicted to cause splice defects with high reliability, it remains difficult to predict whether variants deeper in the intron or those that potentially affect exonic splicing enhancers actually cause splice defects. RNA-seq data, when coupled with variant data, potentially provide a means of correlating variation data with observations of (mis-)splicing patterns.

 

The program fulfils an important need in the community, the results appear promising and will be of special interest to groups performing RNA-seq analysis in medical settings. I have only some minor suggestions that the authors may like to consider.

 

 

Suggestions:

1. The explanation of the methodology is relatively difficult to follow, and I wonder if it might not be better to simplify Figures 1 and 2 for didactic sake. For instance, in Figure 1A, it is unclear where the location of variant *C* is. Does the curved line mean that it could be anywhere in the middle exon?  Also, I assume that exons are being shown in blue and reads shown in gray? 
  Also, the legend text is overly complicated: *D* > *E* swap *D* and *E*. While aficionados of first order logic will follow without problems, I would suggest that it would be better for didactic purposes to delete this and to implicitly assume that *D*< *E* for the sake of this figure. Figure 1B is confusing at this point in the manuscript because the motivation for switching the variable *A*, *B*, *D*, and *E* is not yet clear. On the other hand, panel C and panel D are trivial and do not add anything. I would suggest using Figure 1 to provide one concrete example one a simple level, and stating in the text that the variables are to be switched if the candidate mutation is located on the other side of the exon. 
  Also, the explanations of the method that are couched in first order logic-like notation are difficult to follow, because it is not stated whether the variant *C* can precede the start of the read (in which case *C-S* would be negative). The subscripts for *r* in turn have the subscript s *1* but the variable *S* in the formula does not. 
  Although in the end, I think I follow the overall method, the reader is forced to make arbitrary assumptions in order to interpret the formulae being used to explain the method. A similar comment pertains to the flow chart in Figure 2.
  Therefore, I would suggest the authors take some pains to improve the clarity of the explanation of the method. I would suggest that they show one of two concrete examples and provide English language specifications of the FOL-like formulae that describe the partitioning of reads.
   
2. I am a little unclear on the use of control samples vs experimental samples. Assuming the experimental samples come from different individuals, what is the reason to assume that they will have the same distribution of splice mutations?  And given that one finds dozens of splice variants in normal individuals, what exactly is meant by a control sample? Will control samples not also have lots of splice mutations? How does the method deal with this? And if we are dealing with cancer samples, why not user a paired control to detect cancer-specific mutations? In light of this, the statement " *Maximizing the set of control samples, while computationally more expensive, increases the statistical robustness of the results obtained.",* does not appear to be supported by evidence presented in the manuscript.
   
3. It would be interesting to see a comparison of the distribution of *Ri* values and results of Veridical analysis?
   
4. How does Veridical decide which sequence variant is causative if there are multiple variations located in the vicinity of a given mis-spliced exon? 
   
5. The mutation nomenclature chr1:985377 C>T should not have a space between the position and the nucleotides.
   
6. It is unclear to me why a linear regression model was used to show the performance of the method. The authors could provide timings from real runs.
   
7. It would be interesting to see a plot on the relationship of the p-values called by Veridical and the sequencing depth covered. The authors state " *In particular, a lack of sufficient coverage at a particular locus will cause Veridical to be unable to report any significant results. A coverage of at least 20 reads should be sufficient."*, but they do not provide evidence for this assertion. This is an important question given that low-expressed genes are thus likely to be systematically under-represented in the results of Veridcal, and this should be commented on somewhere in the paper.
   
8. It would be good if the authors provided Sanger validation of at least some of the mis-splicing events reported in the paper.
   
9. The input format for Veridical is described as *"This input format most easily accepts formatted output from the Shannon Pipeline."* Why not allow VCF files and filter them for potential splice variants informatically prior to Veridcal analysis? It was unclear to me how the variants are to be selected and whether Veridical can be easily used outside of the Shannon pipeline?

I have read this submission. I believe that I have an appropriate level of expertise to confirm that it is of an acceptable scientific standard.

# Response

**Published:** 2014-03-27

**Authors:** Peter Rogan

We really appreciate your constructive comments. In particular, we have revised figure 1 as you suggested. Furthermore, we have endeavored to clarify the methods by referencing every corresponding equation pertaining to each informal description of a methodological principle. All references to figures pertain to the first version of the manuscript.

 

- *"It is unclear to me why a linear regression model was used to show the performance of the method. The authors could provide timings from real runs."*
  All data points in Figure 3 (a) consist of data from actual runs. We did not conduct a sufficient number of large-scale runs to accurately determine performance and believe that the regression model provides information for users who wish to use the software in that context.
   
- *"The input format for veridical is described as “This input format most easily accepts formatted output from the Shannon Pipeline.” Why not allow VCF files and filter them for potential splice variants informatically prior to veridcal analysis? It was unclear to me how the variants are to be selected and whether veridical can be easily used outside of the Shannon pipeline?"*
   
- It would be prohibitively difficult for a user and cumbersome to attempt to add such support. The Shannon Pipeline and most other splicing analysis software accept variant call format (VCF) input and outputs their own custom format. VCF is very poorly suited for the annotation of detailed splicing information. The position of the variant is not necessarily the same as the affect splice junction coordinate. While it is possible to label the junction using custom fields in the VCF “INFO” column, the format is not standardized; it would be specific to the particular software used, and thus provides no real advantage over the Shannon pipeline output.
  This would become feasible if the community were to agree upon a standard definition of custom fields; this concept could be generalized to other scenarios in which both a variant and one (or more) other associated coordinates (in this case, a splice site, but elsewhere, perhaps the site of some specific interaction). This process would ultimately require an explicit definition of the content and schema for such additional fields, and would need to solicit views from the community to ensure widespread adoption. We would be interested in contributing to this endeavor.
*"It would be interesting to see a plot on the relationship of the p-values called by Veridical and the sequencing depth covered."  *
  We have conducted a preliminary comparison of p-values for specific evidence types and splicing consequences vs. coverage per exonic base (the coverage of the gene divided by the length of the pertinent exon) and found that the results were quite difficult to interpret. We suspect that this could be explained by the fact that, due to significant intron inclusion, normalizing by exonic length is not really appropriate. Cryptic sites and alternative splicing make direct comparison of these values difficult to compute.
   
- *"How does Veridical decide which sequence variant is causative if there are multiple variations located in the vicinity of a given mis-spliced exon?"*
  Veridical does not currently address cases in which multiple independent variants pertain to the same splice site. In such a case, each variant is analyzed independently.  Based on Mendelian disorders, we have rarely observed multiple independent variants alter the strength of the same splice site, and in any case, the phase of these variants is unknown, so they could reside on different chromosomes. 
   
- *"I am a little unclear on the use of control samples vs experimental samples. Assuming the experimental samples come from different individuals, what is the reason to assume that they will have the same distribution of splice mutations? And given that one finds dozens of splice variants in normal individuals, what exactly is meant by a control sample? Will not control samples also have lots of splice mutations? How does the method deal with this? And if we are dealing with cancer samples, why not user a paired control to detect cancer-specific mutations? In light of this, the statement “Maximizing the set of control samples, while computationally more expensive, increases the statistical robustness of the results obtained.“ Does not appear to be supported by evidence presented in the manuscript."*
  Control samples consist of all non-variant containing (NVC) tumour samples (relative to the variant being analyzed) and normal samples. In the examples shown in the paper, we maximized the number control samples used (106 normal samples and, in general, over 400 NVC samples). Control samples do indeed contain splicing mutations and this is a strength of our statistical paradigm. We term them control samples, because we consider their read distribution to correspond to that expected from the null hypothesis, as they do not contain the specific variant in question. The “noise” introduced by these splicing variants in control samples ensures that we are testing the variant containing samples against samples with many, distinct, splicing mutations. Thus, the greater the number of samples, the smaller the batch effects, which makes the method more reliable. While the normal and tumor DNA sequencing data was paired to call somatic variants, we do not require paired controls for the RNA-Seq data. In fact, TCGA does not provide such data. Since we initially designed the software for the task of analyzing their dataset, we did not wish to assume that paired RNA-Seq data was available.
   
- *"It would be good if the authors provided Sanger validation of at least some of the mis-splicing events reported in the paper."*
  We do not have access to the TCGA tumour samples used. While TCGA performed Sanger validation of a small subset of their variants, none of the variants validated by Veridical had any associated Sanger sequencing data.
   
- *"It would be interesting to see a comparison of the distribution of Ri values and results of Veridical analysis?"*
  The variants predicted by the Shannon splicing mutation analysis pipeline exhibit changes in information content (ΔR _i_). The change in information content of a splice site is directly related to the thermodynamics of the binding event (Schneider, 1997) and therefore the strength of the splice site ( Rogan *et al.,* 1998; Rogan *et al.*, 2003). Therefore, one would expect ΔR _i_ to be highly predictive of valid splicing variants, where cryptic sites are expected to result in increased splice site strength, and natural sites would be expected to be weakened.
  The median ΔR _i _for cryptic variants in the set of pre-Veridical variants (those variants called as somatic mutations and predicted by the Shannon Pipeline to affect mRNA splicing) was 2.6 bits, while the median for validated variants was 2.38 bits. For non-cryptic variants the pre- and post-validation median ΔR _i_ values were -2.52 and -3.18 bits, respectively.
  The information content of cryptic variants actually decreased slightly in the validated set. This likely is related to other factors, such as the initial strength of the natural site and the exon length, which are not accounted for by this analysis. However, the average information content of natural sites did decrease, as expected. The reason for the decrease not being more substantial, again, is likely due to the involvement of other factors, such as the distance of the variant to the natural site – which impacts its R _i_ value. We have refrained from including this discussion in the manuscript, since there is no requirement *per se* for Veridical to be used with the Shannon Pipeline. An explicit definition of the required input format is provided in the paper.

# Peer review: Referee response for version 1

**Published:** 2014-03-07

**Authors:** Liliana Florea

**Affiliations:** 1 McKusick-Nathans Institute of Human Genetics, Johns Hopkins University, Baltimore, MD, USA

The authors describe a method and the associated software, Veridical, for assessing the effects on pre-mRNA splicing of predicted splicing-affecting mutations. To do so the program compares splicing effects, measured by the supporting read counts, in variant-containing (disease) samples against a distribution derived from very large numbers of 'normals', either normal tissue from the same individual or samples from healthy individuals.

The idea is ingenious and novel as applied to mutations affecting splicing, although not in general (see VAAST [ Yandell et al. 2011], which exploits the availability of large numbers of samples to identify likely deleterious variants; it is also the premise for the HapMap and 1000 Genomes projects). The software is fast and practical, being able to test thousands of variants in hundreds of samples within hours. This is the first software of its kind, and if accurate it will be a very valuable resource for clinical genomics.

That being said, while the article provides proof-of-concept and clearly demonstrates the potential of the tool with specific examples, there are several missing pieces that are needed to provide the readers with a view of its overall performance and limitations and to help them use it effectively.

**Major comments:**

1. The article shows numerous positive examples, however there is no indication of the tool's performance in general. The authors should include the results from running the tool on a full data set, to give potential users an idea of the expected outcome.
  Also, several other tools (e.g., MATS, Miso, SpliceTrap) have been developed for the related problem of discovering alternative splicing events and comparing them among samples. MATS in particular, allows differential splicing analyses with multiple replicates. Ideally the paper would include a comparison with MATS on the data set analyzed; this comparison is informative even if MATS is used with only a subset of the samples.
   
2. The method uses the YJ-transformed distribution of supporting read counts across the 'normals' to determine a p-value for the variant, and thus judge its significance and impact on splicing. This is an interesting concept that assumes that with large numbers of 'normals' sample and batch effects will even out; hence, large numbers of samples are required to ensure accuracy. Since these are absolute (non-normalized) counts, however, the method may not work if the variant sample is obtained with a different method, e.g. by rRNA depletion of total RNA whereas most normal samples would come from polyA+ libraries. The authors should clearly discuss this and other possible limitations of their approach.
   
3. Related to the above, the authors mention on several occasions the difficulty in identifying intron inclusion (II) events, in particular the large number of false positives. Indeed, IIs are generally difficult to predict due to the presence of intronic reads ('noise') from unspliced RNA. The levels can vary from sample to sample and across the genome, depending on the sample preparation, gene expression level, splicing efficiency, etc. By comparing read counts exclusively among samples and without taking into account the gene- or genome-level background, Veridical is likely to produce many false positives.
  In particular, the 14 supporting reads in the left intron on Figure 4 seem hardly sufficient to indicate an II event, all the more as there is a larger number of reads in the neighboring intron (not predicted to be II). The authors should provide other type of evidence for this event.
   
4. The mathematical formulas for the various classes of supporting reads and their locations (page 4, continued on page 6) are hard to understand. It would greatly help the readers to include a figure showing schematically the event and read location with respect to the introns and exons.

**Minor comments:**

1. As another reviewer pointed out, the software requires a registration to obtain a temporary license for 30 days, after which the availability and terms of use are unclear. This mode of distribution is not a problem, but the terms should be clearly stated in the manuscript. Also, this is a stand-alone software and not a web tool as implied by the article.
   
2. The authors use the term 'cryptic' splice sites throughout the manuscript (I assume meaning 'aberrantly activated'), but some of the events discovered could be alternative exon ends. It would be helpful to clarify in the context.

This is a potentially very powerful and useful tool. I gave the article an 'Approved with reservation' because it is critical to include results in the aggregate to complement the showcased examples, as well as to discuss its limitations. I will gladly change once these few issues are addressed.

I have read this submission. I believe that I have an appropriate level of expertise to confirm that it is of an acceptable scientific standard, however I have significant reservations, as outlined above.

# Response

**Published:** 2014-03-27

**Authors:** Peter Rogan

We greatly appreciate your remarks and useful suggestions. All references to figures pertain to the first version of the manuscript.

 

First, we address your comments regarding VAAST and *de novo* exome annotation software packages. VAAST uses a feature-based, maximal likelihood, approach to *identify* variants suspected to be pathogenic. While Yandell *et al.* present a general, and indeed very useful method, it is very different from Veridical in two key respects. Most importantly, Veridical does not aim to identify putatively pathogenic variants, but rather, to *confirm* existing *in silico* predictions thereof. Although it is not designed for this purpose, one could envision using the likelihood approach used by VAAST to extract solely splicing variants, annotating them, and then confirming these variants using Veridical with corresponding RNA-Seq data (assuming such data exists). Veridical is the only software available for statistical validation of splicing mutations. While both programs prioritize variants, their goals are rather dichotomous. Similarly, Veridical depends upon the use of an (ideally conservative) exome annotation. We do infer exon skipping events (i.e. alternative splicing) *de novo*, but only to catalog dysregulated splicing “phenotypes” due to genomic sequence variants. Since we rely upon existing annotations, improvements in this area will serve to benefit Veridical and complement it well. In many ways, our parsimonious approach was possible precisely because the problem we address is quite a lot easier to address than *de novo* alternative splicing inference. We are not suggesting that Veridical be used to analyze all variants, resulting from exome or whole-genome sequencing experiments. The Shannon Pipeline, which predicts candidate variants, contains several default or modifiable filters which can limit the number of candidates input to Veridical. We are suggesting that our approach be coupled to existing approaches to act as an *a posteriori*, hypothesis-driven, check on the veridicality of specific variants.

 

We consider alternative splicing to be a different problem, for the following reasons. The authors state: “MATS calculates a *P*-value for each exon isoform by comparing the observed posterior probability with a set of simulated posterior probabilities from the null hypothesis.” We do not simulate posterior probabilities; we calculate them directly from the distribution of control samples, which represent the null hypothesis. Further, MATS requires the user to set uniform thresholds for all alternative splicing events, which in our case, may vary between variants (i.e. inactivating versus leaky mutations). Finally, neither MATS, nor Miso or SpliceTrap provide any means of analyzing novel isoforms created by the activation of cryptic splice sites.

 

We appreciate your insightful summary of our underlying statistical methodology and appreciate the limitations of our statistical inference method. It is certainly the case that the use of different RNA-Seq protocols could prove problematic. Veridical is intended to be used with RNA-Seq data from the same individual as matched DNA-Seq data. We assume that the control RNA-Seq data are generated with comparable sequencing protocols. This has been articulated in the Discussion.

 

We agree that levels of intron inclusion and alternative splicing can be highly variable across samples, due to factors extrinsic to the mutation itself. Such variability is a significant contributory factor to the production of transcriptomic “noise”. Given that statistically significant levels of intron inclusion were observed across all control samples, the prevalence of adjacent intron inclusions (which is not uncommon, *per *se), does not weaken the inference that the predicted splicing mutation increases the levels of intron inclusion.  However, in such cases, the statistical threshold to achieve significance would be higher. A recent analysis indicates that p-value thresholds of 0.005 to 0.001 are appropriate ( Johnson, 2013). Our statistical approach tacitly accounts for this and indeed greater levels of such “noise” could actually increase the statistical utility of our computation. Because of a wide degree of variation of intron inclusion in both normal and tumor transcriptomes, we deliberately elected against normalizing gene level expression in the program.

 

Regarding the specific example cited, the 14 intronic reads in the sample containing the variant  in the read-abundance category (Figure 4 (A)) are contrasted with the per sample mean of 4.051 reads across all non-variant-containing tumours and normal samples in this intron (i.e. the totality of control samples, which results in a p-value of 0.04. We agree that this is not a strong result, and would not permit us to reject the null hypothesis had the threshold significance level been reduced to ≤ 0.01 (which the user is free to stipulate).In this particular case, the variant is still strongly validated, due to the presence of junction-spanning, exon skipping reads (that are absent from all of the control samples). We do not stipulate that the predicted mutation have only one abnormal read type, but rather, that the test assesses each consequence separately. We suggest that the corroborating evidence be taken together in support of variant-induced missplicing. Furthermore, junction-spanning exon skipping is, in general, rarer than intron inclusion in these data. Nevertheless, we do show examples of read-abundance tests for intron inclusion that achieve a significance level of < 0.01, such as within *TMTC2* in Figure 6(C). As you point out, intron inclusion patterns also vary by intron and there are a large number of such intron inclusion reads in the downstream intron in Figure 4(A). However, in our entire set of predicted splicing mutations, we did not find any pertaining to the intron between exons 12 and 13. The observed increased intron inclusion between exons 12 and 13 may be related to the weak (5.2 bit) donor (5’) splice site in exon 12.  In fact, substantial intron inclusion was expected in intron 11, due to the minimal strength of the exon 11 donor of 2.1 bits.

 

Regarding the comment concerning cryptic sites, we always mean non-canonical splice sites activated by sequence-level variation, which are distinctly different from tissue-related alternatively spliced isoforms lacking one or more constitutive exons. We and others have reported numerous examples of common SNPs inducing cryptic splice site activation, which could result in the annotation of accordingly altered transcripts.

 

It is unfortunate that Veridical had to be categorized as a “web tool”. We would have preferred to indicate that the program is a standalone-tool. *F1000Research * does not provide this category, even though other standalone programs have been published by the journal with the Web Tool designation. In the Web Tools category, the journal guidelines require that we provide a set of examples (complete with input and output files) representing the range of results that are obtained with the software. Therefore, providing a full analysis of the complete TCGA breast cancer dataset is beyond the scope of this paper.  It will be presented elsewhere.

 

We believe that the statements in the manuscript's conflict of interests section are sufficient to describe the terms of use. We have added the requested details on Veridical.org, both indicating its duration and instructions for obtaining longer-term access to this software.

# Peer review: Referee response for version 1

**Published:** 2014-01-28

**Authors:** Francesc Xavier Roca

**Affiliations:** 1 Division of Molecular Genetics and Cell Biology, Nanyang Technological University, Singapore, Singapore

This manuscript describes a new computational tool named Veridical, which detects mutant-allele specific splicing changes from large RNAseq datasets. This outstanding tool appears very useful to screen the wealth of transcriptomic data for effects in splicing due to mutations in disease samples, and I think that it will potentially be of interest for many if not all such RNAseq-based studies. In addition, this could spur further efforts to derive similar tools with improved efficiencies. Use of this method should help establish the importance of aberrant splicing in disease as well as the effects of genomic mutations at the RNA level. I only have two comments, that do not diminish my overall rating of this work as of high value:

 

1. I personally disagree with the widespread use of the word “validation” in the title, abstract and text. Authors describe Veridical as a tool to “validate” DNA sequence variants that alter splicing. Indeed, I think that this tool provides an “association” between the variants and splicing, but not a formal proof of their connection. As the genomic and RNA samples usually come from different individuals with many confounding variables, the possibility that the splicing changes arise from factors other than the individual DNA mutations cannot be ruled out. In other words, changes in the levels of trans-acting splicing factors could account in part or totally for the splicing changes across samples. The statistical tests properly conducted in Veridical are designed to minimize such possibility but do not rule it out. In addition, the inherent noisy nature of RNAseq datasets also prompts for caution in the conclusions. To me, the direct proof that a DNA mutation changes splicing of its pre-mRNA can only be provided using minigenes and cell transfection (or in vitro splicing), in which the substrate sequences and cellular context are under almost absolute control. Indeed, the Veridical method is reminiscent of GWAS (Genome-Wide Association Studies), in which the genotype in the DNA, wild-type or mutant, is associated to its phenotype, such as normal versus disease (or other traits) in GWAS, or normal versus aberrant splicing in this study. Thus, for me Veridical provides strong associations – but not validations – between DNA mutations and their effects on splicing.
   
2. As mentioned briefly at the beginning of Discussion, Veridical has built-in prediction tools to prioritize the mutations that are more likely to affect splicing, such as those mapping to splice sites. Even if other sources and tools are cited, a more extensive explanation of these components of Veridical would help the reader/user.

I have read this submission. I believe that I have an appropriate level of expertise to confirm that it is of an acceptable scientific standard.

# Response

**Published:** 2014-03-27

**Authors:** Peter Rogan

We greatly appreciate your review of this paper.

 

Regarding our use of the term “validation”, we understand that *in silico* validation is not comparable to *in vitro* or *in vivo* validation assays. That said, we would like to elaborate. The DNA and RNA samples were matched from the same individual, which is not the case for many *in vitro* assays, i.e. mini-gene expression analysis by reverse transcription followed by PCR. The very large set of controls used in our analysis is also atypical in experimental validation of proposed mutations.  When results are obtained that are statistically significant, it is conventional to refer to them as “validating”. Even if DNA-Seq and RNA-Seq data were not matched from the same individual, Veridical would still determine if known splicing mutations were expressed in a known subset of tumors, however we would instead refer to this as "corroborating".

The comparison with GWAS is inappropriate: we are not comparing means of distinct case and control distributions; rather we are computing the read distribution from the control samples and determining the probability that the mutation bearing sample falls within that distribution. Additionally, the initial hypothesis in a GWAS is quite vague. Thus, the resultant associations are not of the same category as those provided by Veridical. While Veridical does indeed validate the splicing consequence observed, when we say that it validates the mutation we do only mean that it strongly corroborates the mutation as a causative agent of the splicing consequence. The responsibility to decide if the p-value reported by the program is sufficient is left up to the user, who should avoid incorrect *post hoc ergo propter hoc *arguments.

 

Our reference to prioritization of variants for subsequent verification is based upon the result of Veridical's statistical tests. We explicitly mention that the software is not well suited for the analysis of raw output from genome-scale analyses, and that filtering should be performed *a priori*, as we conducted, with a separate Perl script, which is available with Veridical.

# Peer review: Referee response for version 1

**Published:** 2014-01-27

**Authors:** Stefania Bortoluzzi

**Affiliations:** 1 Department of Biology, University of Padova, Padova, Italy

The paper “ *Validation of predicted mRNA splicing mutations using high-throughput transcriptome data*” by Viner *et al*. presents Veridical, a new software for the interpretation and validation of genetic variants identified by DNA sequencing that alter mRNA splicing, leveraging RNA-seq data. The method is based on statistical comparisons of the normalized read counts of abnormally-spliced RNA species in mutant versus non-mutant tissues.

 

Actually, the interpretation of genetic variants is a difficult and key issue in current research.

The integration of genomic and transcriptomic data, namely the use of RNA-seq-based transcriptome characterization as a “molecular phenotype” of cells is useful and meaningful.

 

The software is standalone (not a web-tool) and it is completed by perl scripts, facilitating data management.

The manuscript declare that “Veridical and its associated software programs are available at: www.veridical.org”.

Actually, Veridical is commercially available to the scientific community. A trial version lasting 30 days can be downloaded by the website, but in order to obtain binaries, the website requests a registration with an institutional email address - they reserve the right to deny access to users who register with third-party mail servers (Gmail, Yahoo, Hotmail, etc.).

No pricing information is included in the manuscript and, more importantly, in the webpages accessible to download the software, either before or after registration.

After downloading the software, I was not able to find R scripts that can be useful to generate some plots, as indicated in the manuscript.

 

Saying that, the paper is written in a clear language and it is quite complete.

I propose a few revisions that in my opinion can improve the manuscript readability and clarity.

**Introduction**

 

- Line 13 (minor): indicate which hereditary disease (colon cancer?).

 

**Methods**

 

- 2 ^nd^ Par, Line 5 (minor): “Maximising” is used, but probably the meaning is “increasing” (the number of).
   
- Figure 1 (major): I feel that the info provided by points C and D is trivial, whilst point A's images, sentences and legends can be improved. Figure 1 C and D shows simply examples of reads that are mapped continuously and discontinuously to the reference genome. I think that every potential user of this type of software known well this concept. On the other hand, regarding A and B (upper part of the figure) there is not clear correspondence between the text in the legend and the image, and between the image and the text below (the arch overlap the point A in the figure B, whereas the text says "reads between A and B").
   
- In general, in many cases in the manuscript, the correspondence between legend and figure can be improved, by indicating more clearly the points specific sentences in the legend refer to. Regarding this issue, for instance in Figure 4 I can see indicated neither the “exon 12” nor the “14 reads” mentioned in the legend. Please indicate (using colors, boxes, arrows or overlapping text) key elements in the figure, and revise all figures using the same criterion.
   
- Page 5 (minor): Consider revising the sentence “Furthermore, this transformation allowed us to avoid the use of non-parametric testing, which has its own pitfalls regarding assumptions of the underlying data distribution”, since normally it is assumed that parametric tests ground on assumptions on data distributions, but non-parametric tests by definition can be used without information about data distribution.
   
- End of the next paragraph (major): “It is important to realize, therefore, that the p-values given by Veridical are much more robust when the program is provided with a large number of samples.” This is a pretty clear concept. Please, indicate a general rule to the user/reader: How many samples are required? Setting a reasonable minimum can be more useful for experimental design than saying the larger the sample size the most robust the result.

 

**Results**

 

I have two important criticisms about the Results section:

1. The section is not organized in paragraphs, and mixes performance info (run time using different number of samples and variants) with example results.
   
2. Not clearly saying how these results were obtained. This is important to guarantee repeatability.

 

- (Major) I propose to reorganize the results (considering skipping less important examples; retain surely Fig. 4 and 6) and insert a first paragraph providing information about the dataset used for variants validation (how many samples, how many controls) and about the variant calling (BAM files can be obtained with different settings and criteria and the same apply to calling and filtering of variants). Moreover, please explain how RNA-seq data are treated, and particularly how they are normalized to guarantee cross-samples comparability.
   
- (Major) Also, a brief discussion about the impact of disease samples not carrying the given mutation can be useful, as well as regarding the possibility that a tumour sample not carrying the considered variant can present altered transcriptome since other variants (or factors) impact on the “molecular phenotype”.
   
- Figure 4 (minor): Please comment about the possible existence of intronic transcripts (totally unknown or also annotated in Ensemble, but not displayed in the more conservative RefSeq annotations).
   
- Figure 5 (minor): Please define better the measure “Read−Abundance Total Intron Inclusion”.

I have read this submission. I believe that I have an appropriate level of expertise to confirm that it is of an acceptable scientific standard, however I have significant reservations, as outlined above.

# Response

**Published:** 2014-03-27

**Authors:** Peter Rogan

Thank you for your constructive comments. Regarding the R program, it is embedded within a Perl script to facilitate expeditious parameter and data parsing. All of our figures were generated using the Perl script, which, in-turn, invokes the R script. The program used to generate the histograms is called: VeridicalHist.pl. The invocations of this program are provided in the Perl program documentation on www.veridical.org. We have clarified this in the revision of the manuscript. We will update this web page to include pricing information and details about trial access to the software.

 

The requirement to provide an institutional email address is quite ubiquitous across a number of academic disciplines, including many fields of informatics. In bioinformatics, for example, ANNOVAR requires non-profit users to register an address and HGMD does as well. ChemAxon, software for cheminformatics requires this. In astroinformatics, Sloan Digital Sky Survey III (SDSSIII) also requires an institutional email. These examples are represent a fraction of the numerous scientific websites that request such emails.  We consider it a professional courtesy to provide legitimate email information, and reserve the right to disapprove addresses of individuals who seek to mask their identity, which may enable them to avoid acknowledging the provenance of the software.

 

We specifically address each of your other comments below. All references to figures pertain to the first version of the manuscript.

 

- *"Line 13 (minor): indicate which hereditary disease (colon cancer?)."*
López-Bigas *et al*. (2005) conducted a general analysis using the complete sets of SwissProt genes and OMIM known disease genes. Their mathematical model does not depend upon any specific hereditary disease. Nevertheless, certain genes have been demonstrated to exhibit very high frequencies of splicing mutations ( *ATM*, *NF1*).
   
- *"2 ^nd^ Par, Line 5 (minor): “Maximising” is used, but probably the meaning is “increasing” (the number of)."*
  This has been corrected.
   
- *"Figure 1 (major): I feel that the info provided by points C and D is trivial, whilst point A's images, sentences and legends can be improved. Figure 1 C and D shows simply examples of reads that are mapped continuously and discontinuously to the reference genome. I think that every potential user of this type of software known well this concept. On the other hand, regarding A and B (upper part of the figure) there is not clear correspondence between the text in the legend and the image, and between the image and the text below (the arch overlap the point A in the figure B, whereas the text says "reads between A and B")."*
  This has been revised accordingly.
   
- *"In general, in many cases in the manuscript, the correspondence between legend and figure can be improved, by indicating more clearly the points specific sentences in the legend refer to. Regarding this issue, for instance in Figure 4 I can see indicated neither the “exon 12” nor the “14 reads” mentioned in the legend. Please indicate (using colors, boxes, arrows or overlapping text) key elements in the figure, and revise all figures using the same criterion."*
  In general, we provide exon numbers for reference and to allow for future exon identification. While we specifically describe in the captions which exon contains the variant and relate this to the exon number, we agree that graphical indications of key figure elements within IGV screenshots could improve the clarity. These figures have been revised accordingly.
   
- *"Page 5 (minor): Consider revising the sentence “Furthermore, this transformation allowed us to avoid the use of non-parametric testing, which has its own pitfalls regarding assumptions of the underlying data distribution”, since normally it is assumed that parametric tests ground on assumptions on data distributions, but non-parametric tests by definition can be used without information about data distribution."*
  In this sentence, we are not referring to the ubiquitous assumption of an underlying normal distribution, which is indeed not required for non-parametric tests. Instead, the reference is to, other, lesser known, assumptions that are tacit in most non-parametric methods. The citation we provide (Johnson, 1995) refutes the commonly-held notion that non-parametric tests make no assumptions about the underlying data distribution, and describes numerous pitfalls that can occur when using non-parametric methods. For example, the author articulates a particular assumption implicit in a comparison of means via the Mann-Whitney test which actually requires that, “the two distributions are identical, in shape and scale, differing only in their means. This assumption can be harder to justify than the asymptotic normality demanded by the *t* test, and is rarely evaluated.”
   
- *"End of the next paragraph (major): “It is important to realize, therefore, that the p-values given by Veridical are much more robust when the program is provided with a large number of samples.” This is a pretty clear concept. Please, indicate a general rule to the user/reader: How many samples are required? Setting a reasonable minimum can be more useful for experimental design than saying the larger the sample size the most robust the result."*
  The revised manuscript describes the procedure and criteria for determining the number of control samples needed.
   
- *"The section is not organized in paragraphs, and mixes performance info (run time using different number of samples and variants) with example results."*
  The profiling information and example results are more clearly demarcated in the revision.
   
- *"(Major) I propose to reorganize the results (considering skipping less important examples; retain surely Fig. 4 and 6) and insert a first paragraph providing information about the dataset used for variants validation (how many samples, how many controls) and about the variant calling (BAM files can be obtained with different settings and criteria and the same apply to calling and filtering of variants). Moreover, please explain how RNA-seq data are treated, and particularly how they are normalized to guarantee cross-samples comparability."*
  We now elaborate upon experimental protocols, processing, and data providence. The details of the RNA-Seq data and BAM file generation can obtained from the TCGA consortia, which generated them, specifically in the supplementary methods of Koboldt *et al.* (2012). All of our data input directly into Veridical are available, and even the program used to generate the histograms is provided. This ensures that all of our results are reproducible.
  The input data were obtained by analyzing the original TCGA dataset with the Shannon pipeline, as the paper describes. The pipeline has been published in a peer-reviewed context and is also available on a trial basis.  The details of the TCGA analytical results obtained with the Shannon pipeline are considerable in length, putting them beyond the scope of this paper and will be described elsewhere.
  The journal guidelines concerning “web tools” state: “The article should provide examples of suitable input data sets and include an example of the output that can be expected from the tool and how this output should be interpreted.”  The examples shown are representative of the splicing analysis outcomes present in the full TCGA analysis.  The examples provided in this paper were designed to illustrate the capabilities of Veridical and are not an attempt to forge biological conclusions of this large dataset.
   
- *"(Major) Also, a brief discussion about the impact of disease samples not carrying the given mutation can be useful, as well as regarding the possibility that a tumour sample not carrying the considered variant can present altered transcriptome since other variants (or factors) impact on the “molecular phenotype”."*
  We provide in Table 2 the read counts for non-variant containing tumour samples, and for the variant containing samples. We acknowledge that cancer gene expression results in a gross dysregulation of mRNA splicing thereby causing the presentation of the altered transcriptome. This was a motivating factor behind our choice of statistical method.
   
- *"Figure 4 (minor): Please comment about the possible existence of intronic transcripts (totally unknown or also annotated in Ensemble, but not displayed in the more conservative RefSeq annotations)."*
  If such exons exist, which are annotated as introns by RefSeq, and are actively transcribed in normal or breast cancer tissues, the large number of control samples will reflect this and such events will accordingly not trigger statistically significant intron inclusion events. We have added a comment to the Discussion reflecting this.
   
- *"Figure 5 (minor): Please define better the measure “Read−Abundance Total Intron Inclusion”."*
  This has been addressed in the revision.
